# Health-related quality of life in patients with psoriasis eligible for biologic therapy in southern Iran via the EQ-5D-5 L and DLQI

**DOI:** 10.1038/s41598-026-55839-5

**Published:** 2026-06-11

**Authors:** Ali Mouseli, Maryam Shirvani Shiri, Maryam Tatari, Behzad Raei, Nimetcan Mehmet Orhun, Hassan Karami, Fatemeh Noroozian, Najmeh Bordbar, Sadegh Alishavandi, Mohammad Sadegh Javidan, Omid Zahedi

**Affiliations:** 1 Social Determinants in Health Promotion Research Center, Hormozgan Health Institute, Hormozgan University of Medical Sciences, Bandar Abbas, Iran; 2 Health Sciences Research Center, Torbat Heydariyeh University of Medical Sciences, Torbat Heydariyeh, Iran; 3 Department of Public Health, School of Public Health, Zanjan University of Medical Sciences, Zanjan, Iran; 4 Public Health Department, Faculty of Medicine , Ankara Yildirim Beyazit University, Türkiye; 5 Health Human Resources Research Center, School of Health Management and Information Sciences, Shiraz University of Medical Sciences, Shiraz, Iran; 6 Student Research Committee, Faculty of Medicine, Hormozgan University of Medical Sciences, Bandar Abbas, Iran; 7 Department of Epidemiology and Biostatictics, School of Health, Torbat Heydariyeh University of Medical Sciences, Torbat Heydariyeh, Iran; 8 Social Determinants of Health Research Center, Health and Metabolic Diseases Research Institute, Zanjan University of Medical Sciences, Zanjan, Iran; 9 National Center for Health Insurance Research, Tehran, Iran; 10 Farashband County Healthcare Network, Shiraz University of Medical Sciences, Shiraz, Iran; 11 Student Research Committee, Faculty of Health, Hormozgan University of Medical Sciences, Bandar Abbas, Iran

**Keywords:** Psoriasis, Health-related quality of life, EQ-5D-5 L, DLQI, Biologic therapy, Iran, Diseases, Health care, Medical research

## Abstract

Psoriasis is a chronic inflammatory skin disease that can substantially impair health-related quality of life (HRQoL). While biologic therapies are widely used in patients with psoriasis, their real-world impact on HRQoL within biologic-eligible patient populations remains insufficiently explored, particularly in resource-constrained settings. This cross-sectional study assessed HRQoL in adult patients with psoriasis in Iran who were considered clinically eligible for biologic therapy according to Iranian treatment guideline criteria. HRQoL was assessed via the EQ-5D-5 L, EQ-VAS, and Dermatology Life Quality Index (DLQI). Multiple linear and logistic regression analyses were conducted to identify sociodemographic and clinical factors associated with HRQoL. The mean scores were 0.51 (EQ-5D-5 L), 59.7 (EQ-VAS), and 11.9 (DLQI). A lower HRQoL was significantly associated with greater disease severity, infliximab use, less than six years of education, lack of supplementary insurance, disease duration ≤ 5 years, and being married. Multivariate analysis confirmed the independent effects of these variables. The most commonly reported issues were pain/discomfort (78.5%) and anxiety/depression (75.9%). Patients with psoriasis eligible for biologic therapy in southern Iran experienced poor HRQoL. These real-world findings highlight the need for patient-centered approaches and policy reforms that enhance insurance coverage, improve health literacy, and ensure equitable access to psoriasis care in resource-constrained settings.

**Supplementary Information:**

The online version contains supplementary material available at https://doi.org/10.1038/s41598-026-55839-5.

## Introduction

Psoriasis is a common, chronic, immune-mediated inflammatory skin disease with a relapsing course that affects more than 125 million individuals worldwide^1,2^. In Iran, the estimated prevalence in 2019 was 0.45% (373,112 people)^3^. Owing to its unpredictable nature and profound impact on multiple dimensions of life, psoriasis has consequences that extend far beyond its cutaneous manifestations, posing a complex socioeconomic challenge for both patients and healthcare systems^4,5^.

Psoriasis imposes a substantial burden, with treatment costs reaching 20% of annual income, hospitalization rates reaching 26%, and unemployment affecting approximately half of patients^6^. In addition to physical symptoms, psoriasis can lead to stigma, shame, and psychological distress, adversely affecting patients’ HRQoL^7^. Studies worldwide have consistently demonstrated the physical, social, and psychological burdens of psoriasis, with lower HRQoL scores being more common in females, low-income patients, and those with lower education levels^8,9^. Iranian studies have shown that psoriasis notably affects HRQoL, especially in psychological and social aspects, with disease severity being a major contributing factor—even affecting patients’ families^8,10^.

Today, HRQoL is recognized as a central goal of therapeutic interventions and a crucial tool for planning services and allocating resources. This is especially important for patients with chronic, incurable conditions, as understanding the factors influencing HRQoL plays a vital role in clinical decision-making and in choosing between equally effective treatment options^11,12^. Various instruments are widely used for patient-reported outcomes (PROs) that capture both general and disease-specific aspects of HRQoL, with the EuroQol 5-Dimension (EQ-5D) and Dermatology Life Quality Index (DLQI) being among the most commonly used generic and disease-specific tools^10,13,14^. The EQ-5D-5 L is a standardized generic tool that enables comparisons across diseases and is widely used for utility measurement and economic evaluation^15,16^. The DLQI is a dermatology-specific instrument that assesses the impact of skin disease on various aspects of daily life—including physical symptoms, psychological well-being, social relationships, and occupational functioning—and is considered one of the most validated and widely used tools in dermatology due to its clinical sensitivity and ease of use^17,18^. The combined use of these two instruments provides a more comprehensive assessment of HRQoL by capturing both overall health status and disease-specific impacts.

Although several studies in Iran have assessed the impact of psoriasis on HRQoL, most have been limited to northern and central regions and have focused primarily on general disease features or psychosocial outcomes via the DLQI tool^10,19,20^. In contrast, data on patients from southern regions of the country remain scarce. Given Iran’s geographic, cultural, and economic diversity—and the lack of comprehensive national studies—conducting region-specific research is essential. Moreover, to date, no independent study in Iran has focused on patients eligible for biologic therapy according to clinical guidelines, despite these treatments becoming a cornerstone in the management of moderate-to-severe psoriasis worldwide^21,22^. Considering the high costs and limited accessibility of biologics—especially in underserved areas—evaluating their real-world impact on HRQoL is critical for evidence-based policy-making and equitable resource allocation.

Accordingly, the present study aimed to estimate the HRQoL in patients with psoriasis eligible for biologic therapy according to Iranian clinical guideline criteria via the EQ-5D-5 L and DLQI instruments and to examine its associations with disease severity and duration. In addition, the study assessed the relationships between HRQoL and patients’ demographic and clinical characteristics. The findings from this study may inform policymakers and support HTA for biologic therapies in middle-income settings.

## Methods

### Study design

**Fig. 1:**
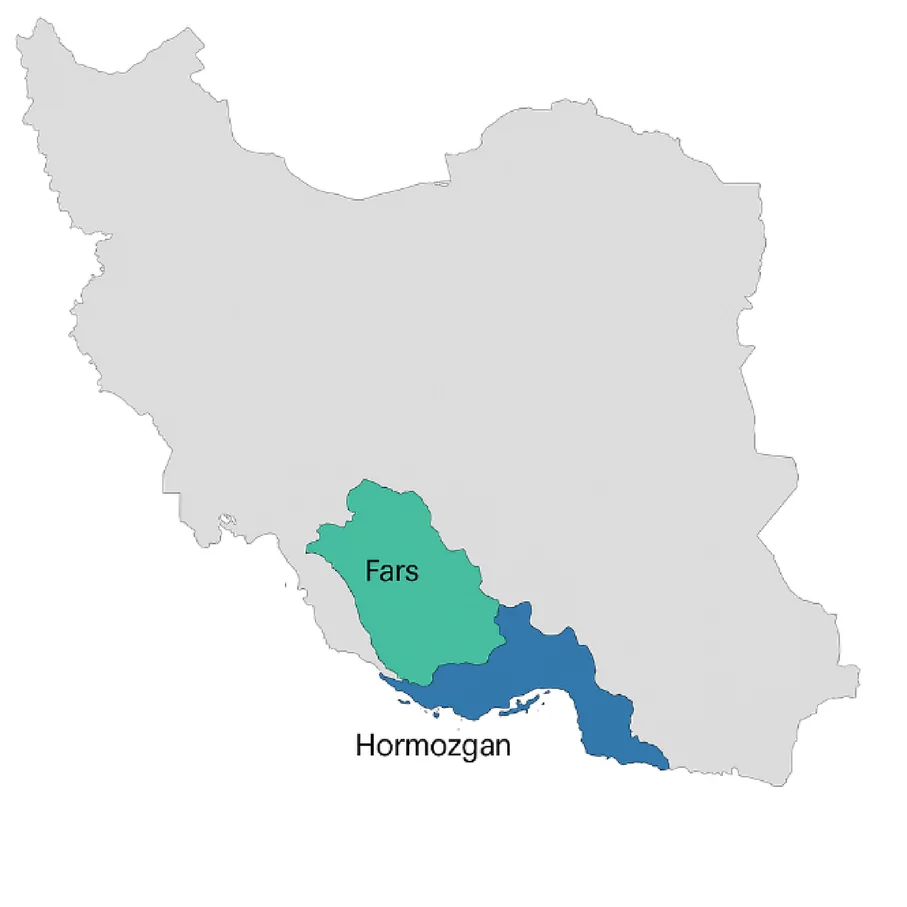
Map of Iran highlighting Fars and Hormozgan provinces as the study area, created with MapChart (24). According to the official information provided by MapChart(https://www.mapchart.net/about.html), all maps generated through this platform are free and distributed under the Creative Commons Attribution-Share Alike 4.0 International (CC BY-SA4.0) license. This license permits the use, modification, and publication of the maps for academicand commercial purposes, provided that appropriate attribution is given.

This cross-sectional study was conducted from October 2024–April 2025 in the southern Iranian provinces of Hormozgan and Fars, encompassing a combined population of 6,627,689 individuals on the basis of the 2016 national census^23^. The geographical locations of these provinces within Iran are illustrated in Fig. 1^24^. The study protocol adhered to the STROBE guidelines for cross-sectional studies^25^.

### Setting and eligibility criteria

In this study, the target population was defined as patients with psoriasis who were clinically eligible for biologic therapy according to Iranian treatment guideline criteria. The participants were adult patients (≥ 18 years) with confirmed diagnoses of psoriasis who had been registered for biologic therapy through either the Social Security Insurance Organization or the Iranian Health Insurance Organization—two major national insurance bodies covering over 90% of the population. Enrollment was based on official insurance records, which require dermatologist-confirmed diagnosis and fulfilment of eligibility criteria for biologic therapy prior to approval of biologic coverage. Individuals were excluded if they were younger than 18 years, had significant physical or mental impairments limiting participation, declined or were unable to provide informed consent. At the time of data collection, participants represented a range of real-world treatment statuses within the biologic-eligible population. While many participants were receiving biologic therapy, a subgroup was receiving nonbiological treatments. These individuals had previously been approved for biologic therapy through national insurance programs but were awaiting treatment initiation, had temporarily discontinued biologic therapy, or had transitioned to alternative treatments owing to clinical or access-related factors. All eligible participants were included to reflect real-world treatment patterns within a cohort of patients with psoriasis eligible for biologic therapy.

### Study outcomes, associated factors, confounders, and diagnostic criteria

#### Outcomes

HRQoL was assessed via three validated instruments: the EQ-5D-5 L index, the EQ-VAS, and the DLQI. Higher scores on the EQ-5D-5 L and EQ-VAS reflect better health status, whereas higher DLQI scores indicate poorer HRQoL.

#### Factors and potential confounders

Sociodemographic and clinical factors included age, sex, marital status, education, employment, place of residence, presence of supplementary insurance, disease severity, illness duration, recent hospitalizations, current treatment status, and comorbidities such as diabetes and rheumatoid arthritis. Age, sex, and comorbid conditions were identified as potential confounders because of their previously established associations with both treatment choice and HRQoL outcomes^9,26^. A category labelled “other treatments” was defined as nonbiologic therapies, primarily comprising targeted systemic agents such as Janus kinase (JAK) inhibitors (e.g., baricitinib) and phosphodiesterase-4 (PDE4) inhibitors (e.g., apremilast), as well as topical therapies, including corticosteroids and vitamin D analogues.

#### Diagnostic criteria

All diagnoses and disease severity assessments were made by board-certified dermatologists. Psoriasis severity was evaluated via the Psoriasis Area Severity Index (PASI), a standardized metric that quantifies both the extent and intensity of disease^27^.

### Data sources and measurement

#### Data sources and collection procedures

Clinical, demographic, and HRQoL data were collected through electronic medical records, structured online questionnaires, and both generic and disease-specific HRQoL instruments. The questionnaires were distributed via widely used messaging platforms (WhatsApp, Telegram, and Eitaa), and the participants recorded their responses themselves. Those with limited digital literacy or without access to these platforms received assistance from trained family members, who were instructed to record the responses exactly as provided by the participant. No responses were completed by healthcare providers or other proxies. Written informed consent was obtained from all participants prior to data collection. To ensure confidentiality, personally identifiable information was anonymized before statistical analysis.

#### Measurement instruments

##### Sociodemographic and clinical characteristics

The variables included age, sex, marital status, educational attainment, employment, urban/rural residence, supplemental insurance status, duration of illness, comorbidities, and treatment regimen.

##### EQ-5D-5 L and EQ-VAS

The validated Persian version of the EQ-5D-5 L was used to assess HRQoL across five domains: mobility (MO), self-care (SC), usual activities (UA), pain/discomfort (P/D), and anxiety/depression (A/D). Each domain was scored on a five-point Likert scale ranging from “no problems” to “extreme problems”^28^. Index values were derived via the Iranian value set developed by Afshari et al., who applied both composite time trade-offs and discrete choice experiment methodologies^29^. The EQ-5D index ranges from − 1.19 to 1, with lower scores indicating poorer health and negative values representing states considered worse than death. The EQ-VAS records participants’ perceived health status on a visual analogue scale ranging from 0 (worst imaginable health) to 100 (best imaginable health)^30^.

##### DLQI

The Persian-validated DLQI questionnaire was employed to evaluate disease-specific HRQoL^17,31^. It consists of 10 questions across six domains: symptoms and feelings (Q1–2), daily activities (Q3–4), leisure (Q5–6), work/school (Q7), personal relationships (Q8–9), and treatment (Q10)^17^. Scores range from 0 to 30, with predefined interpretation bands (0–1 = no effect, 2–5 = small effect, 6–10 = moderate effect, 11–20 = very large effect, 21–30 = extremely large effect)^32^. Internal consistency was verified, with a Cronbach’s alpha of 0.89^31^. A change of ≥ 4 points was considered the minimal clinically important difference (MCID)^33^.

### Ethical considerations

The study protocol received ethical approval from the Ethics Committee of Hormozgan University of Medical Sciences (IR.HUMS.REC.1403.322) in 2024. The DLQI is copyrighted by Cardiff University and was used with permission (licence ID: CUQoL3974).

### Bias minimization

To mitigate selection bias, stratified random sampling was used to ensure provincial representativeness. Standardized and validated instruments were uniformly applied to minimize information bias. Response bias was addressed through follow-up calls and the provision of assistance for participants with low literacy or limited internet access.

### Sample size determination and sampling

#### Descriptive objectives

Sample size estimates were computed to determine the mean HRQoL scores (DLQI, EQ-5D-5 L, and EQ-VAS) with acceptable precision, and previous studies among Iranian psoriasis populations were used as references^8,10,26,31,34,35^. Specifically, the arithmetic mean of reported means and the average of reported standard deviations were used. A margin of error of 10% relative to the mean was adopted, and calculations were conducted via WinPepi (v3.18), assuming a 95% confidence level, 80% statistical power and 10% dropout.

#### Analytical objectives

For subgroup comparisons on the basis of disease severity and duration, effect sizes were calculated via stratified data from previous studies^8,34,35^. Independent t tests and ANOVA were applied accordingly. Sample size calculations were conducted in Stata (v14) via the sampsi command. The maximum required sample size across comparisons was 208 participants.

#### Sampling procedure

Participant lists were obtained from provincial insurance offices. A stratified random sampling approach ensured proportional representation from both provinces. Within strata, patients were randomly selected via computer-generated numbers^36^. In addition to eligibility-based exclusions, some selected individuals were not included due to refusal to participate, invalid or unreachable contact information after repeated follow-up attempts, or incomplete questionnaire responses.

### Handling of quantitative variables

Age and disease duration were categorized into three (< 30, 30–45, and > 45 years) and four (≤ 5, 5–10, 10–18, and > 18 years) groups on the basis of their distributions. Education was grouped into three categories: <6 years, 6–12 years, and > 12 years. Disease severity on the basis of the PASI score was classified as mild (< 7), moderate (7–12), or severe (> 12) following established criteria^37^. Continuous variables were categorized to enhance clinical interpretability and facilitate comparisons with previous studies, as well as to account for potential nonlinear relationships and distributional characteristics of the data.

### Statistical analysis

Continuous variables are reported as the means ± SDs, and categorical variables are reported as frequencies and percentages. Parametric tests (t tests, ANOVA) or nonparametric alternatives (Mann–Whitney U tests, Kruskal–Wallis tests) were applied on the basis of distribution. EQ-5D domain responses were dichotomized into “no problem” and “any problem” and analysed via chi-square tests.

Multiple linear regression was performed to identify factors associated with the EQ-5D index, EQ-VAS score, and DLQI score via a stepwise approach (forward selection and backwards elimination), which was used for exploratory variable selection to derive a parsimonious model given the limited sample size and multiple candidate variables. The stepwise procedure was used for exploratory purposes; therefore, the resulting model should be interpreted with caution and not as a confirmatory model selection approach. Multiple logistic regression was employed for binary EQ-5D dimension outcomes. Model assumptions—including normality, linearity, and the absence of multicollinearity—were verified. Confounders were included in multivariable models on the basis of their theoretical relevance and observed data patterns. Models were built both with and without covariates to test the consistency of the findings. Only 4% of the cases had missing data and were excluded via listwise deletion, which is expected to introduce minimal bias while ensuring a consistent analytic sample across all the models. All analyses were performed in R version 3.6.3, with a significance threshold of *p* < 0.05.

## Results

### Recruitment

**Fig. 2:**
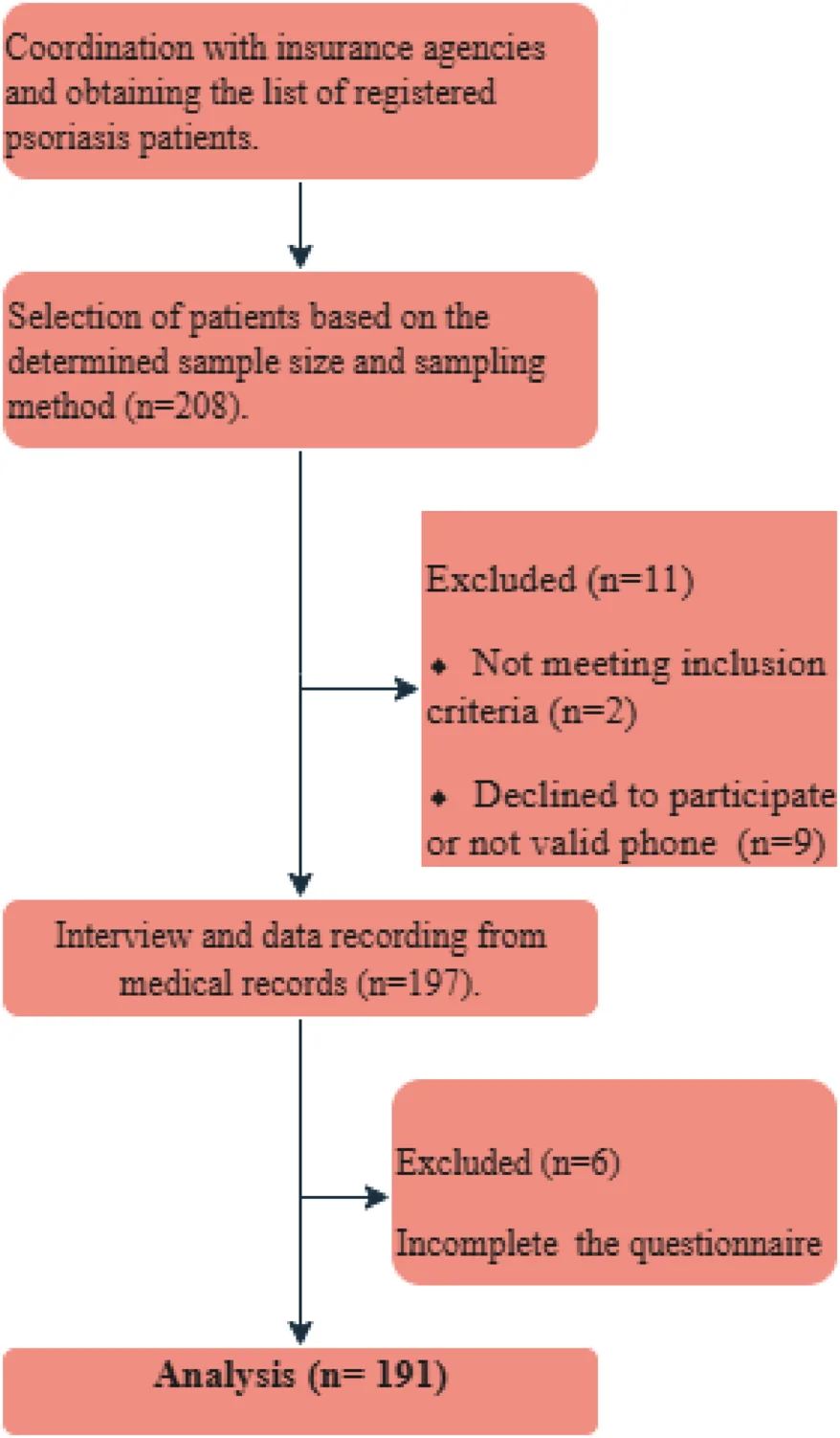
Flowchart of patient recruitment and data collection process.

Among the 208 patients initially selected through the predefined sampling framework, 11 individuals were excluded due to ineligibility, refusal to participate, or invalid/unreachable contact information despite repeated follow-up attempts. An additional 6 participants were excluded because of incomplete questionnaire responses. Consequently, 191 participants were included in the final analysis. A detailed breakdown of the recruitment process is presented in Fig. 2.

### Patient characteristics

Among the 191 included patients, 56% were female, and 70% lived in urban areas. Approximately 41% had severe psoriasis on the basis of the PASI score, and 73% were receiving adalimumab (ADA). Missing data were minimal, with 3% for disease severity and 1% for illness duration, resulting in an overall missing data rate of less than 4% (Table 1; Supplementary Table 1).

**Table 1:** Basic characteristics of psoriasis patients. IFX Infliximab, ADA Adalimumab, Other treatments refer to non-biologic therapies, including targeted systemic agents and topical treatments.

Variables	n(%)
Total	191(100)
Gender	
Male	84(44.0)
Female	107(56.0)
Age groups	
<30	56(29.3)
30-45	86(45.0)
>45	49(25.7)
Residence	
Rural	57(29.8)
Urban	134(70.2)
Marital status	
Single	68(35.6)
Married	123(64.4)
Education	
<6 classes	37(19.4)
6 to 12 classes	88(46.1)
>12 classes	66(34.6)
Sup insurance	
No	139(72.8)
Yes	52(27.2)
Occupation	
Housekeeper	70(36.6)
Unemployed	62(32.5)
Employed	59(30.9)
Drug type	
IFX	21(11.0)
Etanercept	18(9.4)
ADA	140(73.3)
Other treatments	12(6.3)
Hospitalization	
No	162(84.8)
Yes	29(15.2)
Duration of illness	
<=5	48(25.1)
5-10	50(26.2)
10-18	47(24.6)
18>	46(24.1)
Rheumatoid arthritis	
No	170(89)
Yes	21(11)
Diabetes	
No	173(90.6)
Yes	18(9.4)

### Health-related quality of life scores

#### EQ-5D-5 L Index

The mean EQ-5D-5 L index score was 0.51. Significantly higher EQ-5D-5 L scores were observed among patients with mild disease (0.75), those receiving other treatments (0.62), and individuals without a history of hospitalization (0.55). In contrast, participants with comorbid rheumatoid arthritis had markedly lower scores (0.20) (Table 2).

**Table 2:** Mean EQ-5D-5L index, EQ-VAS, and DLQI scores by demographic and clinical characteristics of patients (n = 191).

Variables	EQ-5D-5L index	EQ-VAS score	Total DLQI score
	Mean (SD)	Mean (SD)	Mean (SD)
Total	0.51(0.43)	59.68(25.36)	11.87(8.46)
Gender			
Male	0.51 (0.46)	58.20 (27.01)	12.68(8.43)
Female	0.50 (0.41)	60.83 (24.05)	11.24(8.47)
Age groups			
<30	0.56 (0.40)	62.45 (23.54)	11.38(8.39)
30-45	0.50 (0.43)	59.15 (26.21)	13.21(7.86)
>45	0.455 (0.46)	57.42 (26.08)	10.10(9.27)
Residence			
Rural	0.50 (0.38)	59.21 (22.66)	11.74(7.35)
Urban	0.51 (0.45)	59.87 (26.51)	11.93(8.90)
Marital status			
Single	**0.61 (0.33)**	63.29 (24.07)	**10.01(7.13)**
Married	0 .45(0.47)	57.67 (25.93)	12.90(8.96)
Education			
<6 classes	0.33 (0.47)	50.43 (26.69)	12.41(7.83)
6 to 12 classes	0.50 (0.42)	58.69 (25.53)	13.06(9.04)
>12 classes	**0.61 (0.39)**	**66.17 (22.82)**	10.00(7.74)
Sup insurance			
No	0.49 (0.42)	58.33 (25.66)	12.89(8.55)
Yes	0.56 (0.47)	63.27 (24.41)	**9.15(7.61)**
Occupation			
Housekeeper	0.44 (0.44)	58.19 (24.59)	12.21(8.88)
Unemployed	0.49 (0.42)	57.36 (25.84)	12.45(8.18)
Employed	**0.61 (0.42)**	63.89 (25.67)	10.86(8.26)
Disease severity			
Mild	**0.75 (0.39)**	**75.56 (25.62)**	**5.20(7.08)** *
Moderate	0.55 (0.42)	61.52 (22.50)	10.42(7.37)
Severe	0.38 (0.42)	52.50 (25.92)	15.65(8.24)
Drug type			
IFX	0.19 (0.56)	49.71 (29.92)	16.19(8.69)
Etanercept	0.39 (0.40)	65.17 (21.21)	14.33(8.54)
ADA	0.56 (0.40)	59.86 (25.04)	11.02(8.23)
Other treatments	**0.62 (0.34)**	**66.66 (23.87)**	10.58(8.47)*
Hospitalization			
No	**0.55 (0.38)**	59.94 (24.76)	11.24(8.26)*
Yes	0.26 (0.59)	58.21 (28.91)	15.41(8.78)
Duration of illness			
<=5	0.43 (0.54)	61.83 (26.08)	11.65(8.73)
5-10	0.47 (0.43)	56.36 (26.75)	11.90(9.06)
10-18	0.56 (0.31)	60.74 (24.93)	13.30(7.58)
18>	0.57 (0.40)	59.94 (23.92)	10.63(8.38)
Rheumatoid arthritis			
No	0.54(0.40)	59.08(24.88)	11.73(8.27)
Yes	**0.20(0.55)**	64.48(29.21)	13.05(9.99)
Diabetes			
No	0.49(0.44)	59.62(25.56)	12.31(8.48)
Yes	0.64(0.26)	60.22(24.09)	**7.67(7.20)**

Boldness statistically significant differences, * Clinically significance, EQ-5D-5L Five level of Euro QoL Five-Dimension Health Questionnaire, EQ-VAS EuroQol Visual Analogue Scale, SD Standard Deviation, Sup Insurance Supplementary Insurance, IFX Infliximab, ADA Adalimumab, Other treatments refer to non-biologic therapies, including targeted systemic agents and topical treatments.

#### EQ-VAS score

The mean EQ-VAS score was 59.7. The participants with mild psoriasis reported significantly higher scores (75.6), followed by those with higher education (66.2) and those receiving other treatments (66.6). Conversely, patients treated with infliximab (IFX) reported the lowest mean EQ-VAS score, at 49.7 (Table 2).

#### Total DLQI score

The mean DLQI score was 11.9. Patients with severe psoriasis reported the highest scores (15.7), whereas those with mild disease had the lowest scores (5.2); both differences were clinically and statistically significant. Significantly lower DLQI scores were observed among patients who were single, had supplementary insurance, had no history of hospitalization, and were receiving other treatments (Table 2).

### Associated factors of health-related quality of life

#### EQ-5D-5 L index

In the multivariable linear regression analysis, mild (β = 0.35; 95% CI: 0.16, 0.53) and moderate disease (β = 0.13; 95% CI: 0.004, 0.25) were significantly positively associated with the EQ-5D-5 L score compared with severe psoriasis. In contrast, treatment with IFX (β = − 0.43; 95% CI: − 0.71, − 0.15) and being married (β = − 0.13; 95% CI: − 0.25, − 0.01) were significantly negatively associated with the index scores (Table 3).

**Table 3:** Multiple linear regression associated between demographic/clinical characteristics and EQ-5D-5L index, EQ-VAS score and total DLQI score.

Determinants	EQ-5D-5L index			EQ-VAS			DLQI		
	β coef. (95% CI)	P value	STZ β	β coef. (95% CI)	P value	STZ β	β coef. (95% CI)	P value	STZ β
Marital status									
Married	-0.13(-0.25, -0.01)	0.033	-0.14	–	–	–	2.98(0.77, 5.20)	0.009	0.17
Single (Ref.)									
Education									
<6 classes	-0.19(-0.36, -0.02)	0.026	-0.17	-13.07(-23.03, -3.11)	0.010	-0.21	–	–	–
6 to 12 classes	-0.04(-0.17, 0.10)	0.609	-0.04	-4.70(-12.59, 3.19)	0.241	-0.09	–	–	–
>12 classes (Ref.)									
Sup insurance									
No (Ref.)									
Yes	–	–	–	–	–	–	-4.20(-6.60, -1.81)	0.001	-0.22
Disease severity									
Mild	0.35(0.16, 0.53)	0.001	0.27	21.36(10.27, 32.44)	0.001	0.29	-9.94(-13.24, -6.63)	0.001	-0.40
Moderate	0.13(0.004, 0.25)	0.043	0.15	7.07(-0.50, 14.62)	0.067	0.14	-4.57(-6.82, -2.31)	0.001	-0.27
Severe (Ref.)									
Drug type									
IFX	-0.43(-0.71, -0.15)	0.003	-0.31	–	–	–	5.86(0.65, 11.06)	0.019	0.22
Etanercept	-0.26(-0.55, 0.03)	0.074	-0.18	–	–	–	5.17(-1.18, 10.52)	0.18	0.18
ADA	-0.04(-0.27, 0.19)	0.735	-0.04	–	–	–	0.60(-3.74, 4.93)	0.456	0.03
Other (Ref.)									
Disease duration									
<=5	-0.17(-0.33, -0.01)	0.039	-0.17	–	–	–	–	–	–
5-10	-0.06(-0.22, 0.10)	0.491	-0.06	–	–	–	–	–	–
10-18	0.02(-0.14, 0.12)	0.794	0.02	–	–	–	–	–	-
18> (Ref.)									

EQ-5D-5L Five level of EuroQol five-dimension health questionnaire, EQ-VAS EuroQol visual analogue scale, DLQI dermatology life quality index questionnaire, SD standard deviation, Sup insurance supplementary insurance, IFX infliximab, ADA adalimumab, other treatments refer to non-biologic therapies, including targeted systemic agents and topical treatments.

#### EQ-VAS score

Patients with mild psoriasis reported significantly higher EQ-VAS scores (β = 21.36; 95% CI: 10.27, 32.44). In contrast, participants with fewer than six years of education had significantly lower scores (β = − 13.07; 95% CI: − 23.03, − 3.11) (Table 3).

#### DLQI total score

Compared with those with severe disease, patients with mild and moderate psoriasis presented significantly lower DLQI scores by 9.94 points (β = − 9.94; 95% CI: − 13.24, − 6.63) and 4.57 points (β = − 4.57; 95% CI: − 6.82, − 2.31), respectively. Having supplementary insurance was also significantly associated with lower DLQI scores (β = − 4.20; 95% CI: − 6.60, − 1.81). In contrast, treatment with IFX was associated with significantly higher DLQI scores (β = 5.86; 95% CI: 0.65, 11.06), indicating greater impairment (Table 3).

### Reported problems in the EQ-5D-5 L dimensions

**Fig. 3:**
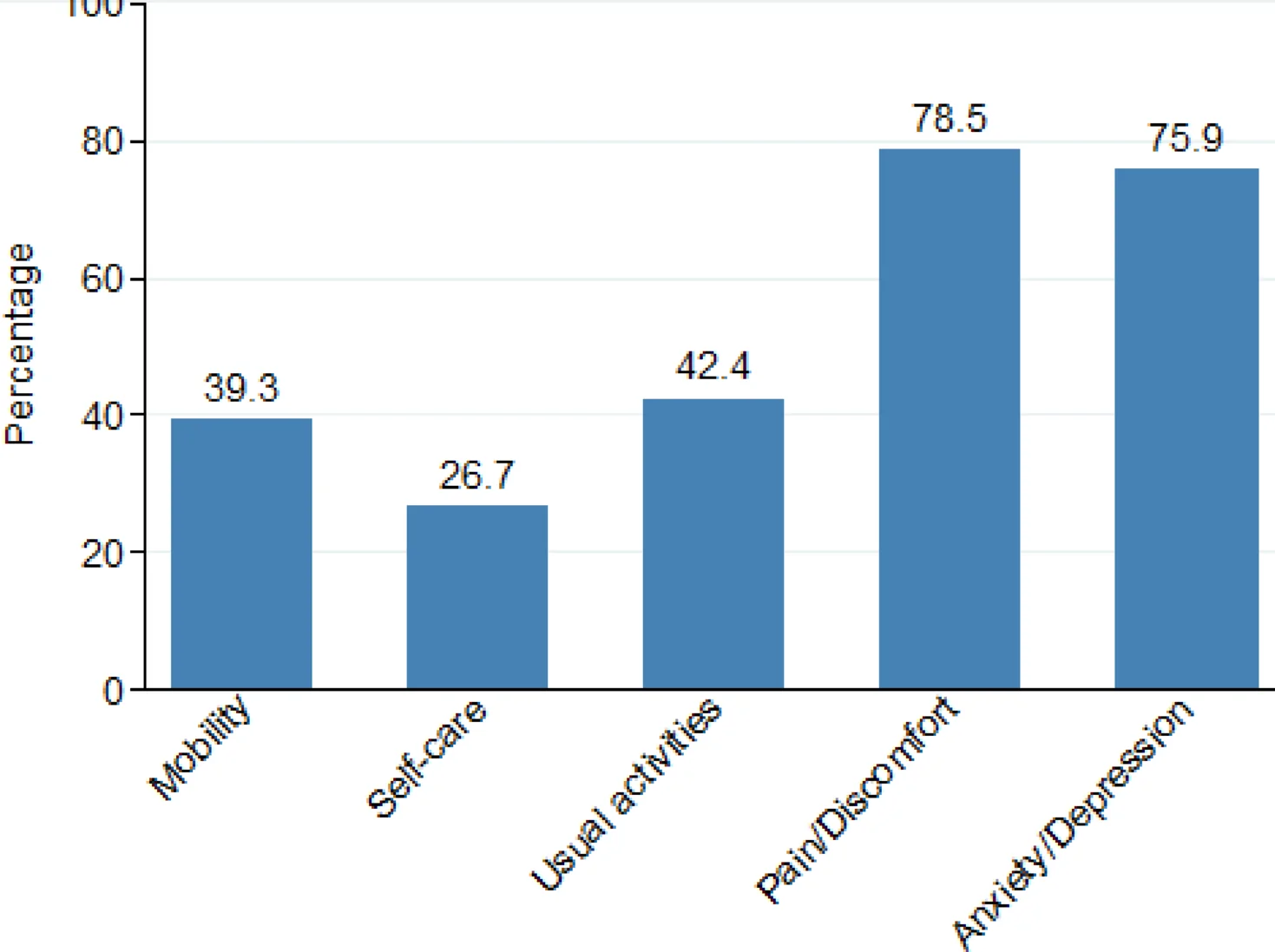
Proportion of patients reporting any problem across EQ-5D-5 L dimensions.

The highest proportions of reported problems were observed in the domains of P/D (78.5%) and A/D (75.9%), followed by UA (42.4%) and MO (39.3%). SC was the least affected dimension, with only 26.7% of patients reporting difficulties (Fig. 3).

### Factors associated with the EQ-5D dimensions

Logistic regression models demonstrated that severe psoriasis and comorbid rheumatoid arthritis were significantly associated with increased odds of reporting problems in multiple EQ-5D dimensions. In contrast, mild disease and other treatments were linked to reduced odds of impairment across several domains (Supplementary Table 2).

### Associated factors of impairment across DLQI dimensions

Multivariate regression analysis revealed that patients with severe psoriasis experienced significantly greater impairments across all six DLQI domains. Furthermore, treatment with IFX was significantly associated with increased impairment scores in multiple domains (Supplementary Table 3).

## Discussion

Overall, patients with psoriasis who were clinically eligible for biologic therapy in southern Iran demonstrated poor HRQoL. The greatest impairments were observed in those with severe disease, IFX treatment, less than six years of formal education, no supplementary insurance, and disease duration under five years. This study provides real-world evidence on the HRQoL of patients treated with biologics in a lower-middle-income setting, where formal clinical trial data may be limited.

Overall HRQoL status in the study population was notably suboptimal across all measurement instruments. The mean EQ-5D-5 L index was 0.51, and the average EQ-VAS score was 59.68, both of which align with the lower ends of internationally reported ranges^10,13,38^. Similarly, the mean DLQI score of 11.87 indicated a very large effect on HRQoL, which is consistent with findings from Iran and other countries^8,14,31,39^. These results reflect the considerable burden of psoriasis compared with the general Iranian population and other chronic disease groups^40–42^. Variations in value sets, national social health policies, healthcare system characteristics, and sociocultural factors may contribute to these differences, although cross-country comparisons should be interpreted with caution given the contextual differences and the national scope of this study.

Disease severity demonstrated a robust association with all HRQoL measures, as confirmed by both unadjusted group comparisons and multiple linear regression. Patients with milder psoriasis reported higher EQ-5D, EQ-VAS, and DLQI scores, which is consistent with the findings of previous studies^26,34,39,43^. The difference in DLQI scores was both statistically and clinically significant, whereas differences in EQ-5D scores and EQ-VAS scores, although lacking established clinical thresholds, were statistically meaningful. A few studies have reported no such associations^43–45^, possibly due to methodological or contextual variations.

Disease duration was modestly associated with HRQoL, primarily through its impact on the EQ-5D index. Our findings revealed a significant negative association between shorter disease duration (≤ 5 years) and EQ-5D scores—a pattern that is consistent with previous studies highlighting the elevated burden in early-stage psoriasis patients^14,35,39,46^. In contrast, other studies have reported no significant associations^20,47–49^. It is plausible that patients in earlier stages of the disease experience greater psychosocial distress, whereas those with longer disease durations may develop adaptive coping strategies that buffer the impact on HRQoL.

Our findings indicate that the current type of biologic therapy is associated with differential HRQoL outcomes. Patients receiving IFX reported the lowest HRQoL scores across all three instruments, with statistically significant differences and clinically meaningful reductions, particularly in the DLQI. These associations were confirmed through multiple linear regression analyses for both the EQ-5D and DLQI and are consistent with previous studies^10,48^. In contrast, patients in the “other therapies” group reported higher HRQoL scores, likely reflecting less severe disease and a shorter disease duration, as biologics are generally prescribed for more severe cases. Given the cross-sectional design of the study, these differences reflect correlations rather than causal effects. The observed differences may also be influenced by confounding by indications, whereby baseline clinical characteristics such as initial disease severity, comorbidities, and prior treatment failures could explain variations in HRQoL across therapies. Therefore, comparisons between treatments should be interpreted cautiously, accounting for these underlying differences. These findings highlight that HRQoL among patients is influenced by multiple clinical and demographic factors and that individual patient experiences can vary widely. Moreover, understanding these HRQoL differences between treatment patterns can guide patient-centered decision-making and provide evidence-based recommendations to HTA patients and payers to optimize the use of biologic therapies in resource-constrained settings.

Higher educational attainment was significantly associated with better HRQoL, especially for the EQ-5D and EQ-VAS scores. Patients with more than 12 years of education had the highest scores (0.61 and 66.17), significantly exceeding those with less education. Regression analysis confirmed these associations. However, some studies reported no significant associations, which may be due to differences in sample characteristics or insufficient adjustment for key confounders^46,48^. This finding supports prior evidence linking higher education to better health literacy and engagement, underscoring the need for supportive measures for lower-educated populations^35^.

Marital status was significantly associated with the EQ-5D and DLQI scores according to both bivariate and multiple linear regression analyses. Single patients reported better HRQoL than married patients did. While some studies have linked marriage to increased psychosocial burden and reduced HRQoL^20,47^, others have associated marriage with emotional support and better outcomes^46,49^, and some have reported no significant associations^44,48,50^. These discrepancies may reflect differences in culture, measurement tools, or study design. Investigating mediating factors such as caregiving, financial stress, and social support may clarify these patterns and guide targeted public health strategies.

Supplementary health insurance was positively and significantly associated with HRQoL, as measured by the DLQI, in both bivariate and multivariable analyses. This finding is consistent with prior studies^14,31^. The results are relevant for HTA agencies and policymakers involved in resource allocation and reimbursement decisions for high-cost biologic therapies in middle-income countries.

In bivariate analyses, diabetes, rheumatoid arthritis, and hospitalization history were each significantly negatively associated with HRQoL, and employment status was significantly positively associated—but these associations were observed only for a single HRQoL instrument in each case. None of the variables remained significant in the multiple linear regression models, likely due to the influence of other dominant clinical or sociodemographic factors. Further investigations of these variables in larger and more targeted studies are recommended.

Dimension-level analyses of HRQoL revealed that greater disease severity, rheumatoid arthritis, and biologic therapies were significantly associated with increased odds of reporting problems with MO, SC, UA, and P/D. In the DLQI domain analysis, disease severity was significantly associated with all dimensions. Additionally, a lack of supplementary insurance, IFX use, and diabetes status were negatively and significantly associated with multiple HRQoL dimensions. These findings are broadly consistent with those of prior studies^9,19,50^, although some discrepancies across the literature—possibly due to differences in health system structures and patient expectations—highlight the need for further investigations in more diverse populations^9,43^.

### Strengths and limitations

This study has several methodological strengths. The use of two validated instruments—a generic instrument (EQ-5D-5 L) and a dermatology-specific instrument (DLQI)—allowed for a comprehensive assessment of HRQoL in patients with psoriasis. In addition, the relatively large sample size provided sufficient statistical power to detect meaningful associations in the primary analyses.

However, several limitations should be acknowledged. First, the cross-sectional design of the study precludes causal interpretations and limits the ability to assess temporal or long-term changes in HRQoL. Second, although the overall sample size appeared adequate, it may have been insufficient to detect statistically significant differences in certain specific dimensions of HRQoL. Third, the study population was drawn from insurance-approved patients in a single region in southern Iran, which may limit the generalizability of the findings to the broader psoriasis population. In addition, the absence of qualitative or mixed-methods data is another notable limitation, as such approaches could have provided deeper insights into patients’ perceptions and experiences regarding treatment patterns. Moreover, the sampling frame was based on institutional insurance registries and telephone accessibility, which may have preferentially included individuals with greater access to healthcare services. Consequently, patients with limited healthcare access or without insurance coverage may be underrepresented, potentially affecting the generalizability of the findings. For patients with limited digital literacy or access, trained family members assisted in recording responses, which may introduce potential information bias, including differences in the interpretation of items or social desirability effects. In addition, the use of stepwise regression should be interpreted as exploratory rather than confirmatory and may be sensitive to sample-specific variation.

## Conclusion

This study demonstrated that many patients with psoriasis in southern Iran, including many who were receiving such treatments, experience poor HRQoL despite being clinically eligible for biologic therapy. Factors such as greater disease severity, current IFX treatment, fewer than 12 years of education, lack of supplementary insurance, shorter disease duration (≤ 5 years), and being married were significantly associated with lower HRQoL. Our real-world evidence highlights the need for patient-centered care models tailored to the socioeconomic context.

## Supplementary Information

Below is the link to the electronic supplementary material.


Supplementary Material 1


## Data Availability

The datasets used and/or analyzed during the current study are available from the corresponding author on reasonable request.
