# Supplementary material for: Health-related quality of life in patients with psoriasis eligible for biologic therapy in southern Iran via the EQ-5D-5 L and DLQI
Source: Sci Rep. 2026 Jun 11;16:27495. doi: 10.1038/s41598-026-55839-5 (PMC13534552; doi:10.1038/s41598-026-55839-5)
Supplement: Supplementary file 1 [file 41598_2026_55839_MOESM1_ESM.docx]

**Supplementary Table 1.** Distribution of disease severity

and disease effect on patients’ HRQoL.

| Variable |  | n(%) |
| --- | --- | --- |
| Psoriasis severity  based on PASI | Mild | 25(13.1) |
|  | Moderate | 88(46.1) |
|  | Severe | 78(40.8) |
|  | Total | 191(100) |
| Disease effect on patient's HRQoL based on DLQI | No effect | 20(10.5) |
|  | Small effect | 34(17.8) |
|  | Moderate effect | 39(20.4) |
|  | Very large effect | 62(32.5) |
|  | Extremely large effect | 36(18.8) |
|  | Total | 191(100) |

HRQoL Health-related Quality of Life, PASI Psoriasis Area Severity
Index, DLQI Dermatology Life Quality Index Questionnaire.

**Supplementary Table 2.** Variables associated with “any problems” in each EQ-5D 5L dimension using multiple logistic regression.

| **Variables** | **Odds ratio** | | | | |
| --- | --- | --- | --- | --- | --- |
|  | MO | SC | UA | P/D | A/D |
| **Marital Status** |  |  |  |  |  |
| Single **(ref.)** |  | | | | |
| Married | - | **6.23(1.69, 19.79)** | **2.69(1.18, 6.11)** | - | - |
| **Sup insurance** |  |  |  |  |  |
| No **(ref.)** |  | | | | |
| Yes | **-** | - | **0.24(0.09, 0.58)** | - | - |
| **Occupation** |  |  |  |  |  |
| Housekeeper | **5.31(1.73, 16.30)** |  |  |  |  |
| Unemployed | **2.56(1.10, 5.65)** |  |  |  |  |
| Employed **(ref.)** |  | | | | |
| **Disease Severity** |  |  |  |  |  |
| Mild | **0.10(0.03, 0.41)** | **0.11(0.02, 0.62)** | **0.10(0.03, 0.41)** | **0.19(0.07, 0.54)** | **0.23(0.09, 0.63)** |
| Moderate | 0.54(0.27, 1.07) | 0.50(0.10, 0.68) | **0.31(0.14, 0.68)** | 1.24(0.54, 2.87) | 0.74(0.35, 1.59) |
| Severe **(ref.)** |  | | | | |
| **Drug Type** |  |  |  |  |  |
| IFX | **12.18(1.80, 83.33)** | **35.45(2.17, 500)** | **26.65(2.69, 250.0)** | - | - |
| Etanercept | **10.68(1.51, 76.92)** | **79.38(4.22, 980)** | **22.37(2.39, 200.0)** | - | - |
| ADA | **4.89(0.90, 26.32)** | **9.42(0.70 – 111)** | **8.52(1.24, 52.61)** | - | - |
| Other treatments **(ref.)** |  | | | | |
| **Hospitalization** |  |  |  |  |  |
| No **(ref.)** |  | | | | |
| Yes | - | - | **3.94(1.21, 13.2)** | - | - |
| **Duration of illness** |  |  |  |  |  |
| <=5 | - | **4.90(1.45, 14.3)** | **4.08(1.30, 12.82)** | 1.81(0.67, 4.93) | - |
| 5-10 | - | 3.09(0.94, 9.43) | **2.84(1.03, 8.33)** | 2.56(0.92, 7.14) | - |
| 10-18 | - | 0.94(0.27, 3.36) | 1.23(0.44, 3.70) | **3.60(1.18, 10.99)** | - |
| 18> **(ref.)** |  | | | | |
| **Rheumatoid arthritis** |  |  |  |  |  |
| No **(ref.)** |  | | | | |
| Yes | **3.91(1.28, 11.94)** | **12.02(2.88, 42.47)** | **9.98(2.70, 50.0)** | **9.95(0.01, 0.86)** | - |

**Boldness** statistically significant differences, Ref, reference group, Other treatments refer to non-biologic therapies, including targeted systemic agents and topical treatments. Note: Some odds ratios may be unusually large due to small cell counts or model instability. Interpret these estimates with caution.

**Supplementary Table 3.** Associations between the DLQL dimensions, and demographic/clinical characteristics in psoriatic patients. (n = 191).

| **Variables** | **DLQI Dimensions Mean (SD)** | | | | | |
| --- | --- | --- | --- | --- | --- | --- |
|  | **Symptom**  **and feelings** | **Daily**  **activities** | **Leisure** | **Work and**  **school** | **Personal**  **relationships** | **Treatment** |
| **Total** | 3.14(1.90) | 2.50(1.96) | 2.35(2.00) | 1.06(1.34) | 1.80(2.05) | 1.03(0.99) |
| **Gender** |  |  |  |  |  |  |
| Male | 3.15(1.88) | 2.51(1.96) | 2.65(2.05) | 1.15(1.30) | 2.08(2.13) | 1.12(1.03) |
| Female | 3.13(1.92) | 2.49(1.97) | 2.10(1.942) | 0.99(1.370) | 1.58(1.967) | 0.95(0.95) |
| **Age Groups** |  |  |  |  |  |  |
| <30 | 3.29(2.04) | 2.50(1.98) | 2.14(2.07) | 0.93(1.31) | 1.57(1.896) | 0.95(0.98) |
| 30-45 | 3.23(1.77) | 2.70(1.91) | **2.90(1.90)** | 1.16(1.34) | 2.09(2.12) | 1.13(0.94) |
| >45 | 2.82(1.95) | 2.14(2.02) | 1.61(1.86) | 1.04(1.39) | 1.55(2.08) | 0.94(1.06) |
| **Residence** |  |  |  |  |  |  |
| Rural | 3.12(1.61) | 2.32(1.71) | 2.35(1.88) | 1.04(1.33) | 1.88(2.03) | 1.04(0.92) |
| Urban | 3.15(2.01) | 2.57(2.06) | 2.34(2.06) | 1.07(1.34) | 1.77(2.06) | 1.02(1.01) |
| **Marital Status** |  |  |  |  |  |  |
| Single | 2.79(1.86) | 2.35(1.91) | 2.00(1.89) | 0.99(1.31) | 1.06(1.41) | 0.82(0.84) |
| Married | **3.33(1.89)** | 2.58(1.99) | 2.54(2.04) | 1.11(1.36) | **2.21(2.23)** | 1.14(1.04) |
| **Education** |  |  |  |  |  |  |
| <6 Classes | **3.43(1.78)** | 2.70(2.02) | 2.38(2.01) | 1.05(1.39) | 1.76(1.86) | 1.08(1.01) |
| 6 to 12 Classes | **3.47(2.00)** | 2.69(1.99) | 2.48(2.01) | 1.19(1.38) | 2.08(2.21) | 1.15(1.01) |
| >12 Classes | 2.55(1.69) | 2.12(1.86) | 2.15(1.99) | 0.89(1.25) | 1.45(1.88) | 0.83(0.921) |
| **Sup insurance** |  |  |  |  |  |  |
| No | 3.28(1.90) | **2.71(2.02)** | **2.65(2.04)** | 1.14(1.38) | **1.96(2.08)** | **1.14(1.02)** |
| Yes | 2.77(1.84) | 1.92(1.66) | 1.52(1.62) | 0.85(1.19) | 1.37(1.90) | 0.73(0.819) |
| **Occupation** |  |  |  |  |  |  |
| Housekeeper | 3.40(1.90) | 2.61(1.98) | 2.20(1.95) | 1.04(1.39) | 1.90(2.14) | 1.06(0.976) |
| Unemployed | 3.03(1.95) | 2.68(2.06) | 2.66(2.06) | 1.23(1.40) | 1.79(1.92) | 1.06(0.990) |
| Employed | 2.95(1.83) | 2.17(1.81) | 2.19(1.99) | 0.92(1.193) | 1.69(2.08) | 0.95(1.00) |
| **Disease Severity** |  |  |  |  |  |  |
| Mild | 1.40(1.50) | 1.00(1.47) | 1.08(1.86) | 0.44(1.00) | 0.80(1.58) | 0.48(0.87) |
| Moderate | 2.85(1.66) | 2.22(1.65) | 2.15(1.85) | 0.86(1.22) | 1.48(1.95) | 0.86(0.899) |
| Severe | **4.03(1.78)** | **3.29(2.07)** | **2.97(1.99)** | **1.49(1.43)** | **2.49(2.08)** | **1.38(0.99)** |
| **Drug Type** |  |  |  |  |  |  |
| IFX | 3.95(1.71) | **3.57(2.15)** | **3.24(2.11)** | **1.81(1.43)** | 2.14(2.265) | 1.48(1.16) |
| Etanercept | 2.94(2.07) | 3.28(1.84) | 3.11(2.05) | 1.44(1.46) | 2.39(2.20) | 1.17(0.924) |
| ADA | 3.03(1.85) | 2.26(1.87) | 2.12(1.92) | 0.93(1.27) | 1.71(2.04) | 0.97(0.944) |
| Other treatments | 3.33(2.30) | 2.25(2.09) | 2.25(2.13) | 0.75(1.35) | 1.33(1.43) | 0.67(1.07) |
| **Hospitalization** |  |  |  |  |  |  |
| No | 3.08(1.90) | 2.37(1.89) | 2.19(1.92) | 0.97(1.30) | 1.67(1.97) | 0.96(0.94) |
| Yes | 3.48(1.88) | 3.21(2.22) | **3.24(2.23)** | **1.59(1.42)** | 2.52(2.33) | 1.38(1.14) |
| **Rheumatoid arthritis** |  |  |  |  |  |  |
| No | 3.07(1.87) | 2.46(1.91) | 2.29(1.98) | 1.08(1.34) | 1.79(2.02) | 1.04(0.98) |
| Yes | 3.71(2.05) | 2.81(2.38) | 2.76(2.21) | 0.95(1.36) | 1.86(2.35) | 0.95(1.07) |
| **Diabetes** |  |  |  |  |  |  |
| No | 2.28(1.81) | 1.67(1.81) | 1.17(1.25) | 0.89(1.37) | 0.67(1.19) | 1.00(0.97) |
| Yes | **3.23(1.89)** | 2.58(1.96) | **2.47(2.03)** | 1.08(1.34) | **1.92(2.09)** | 1.03(0.99) |
| **Disease Duration** |  |  |  |  |  |  |
| <=5 | 3.08(1.94) | 2.38(1.89) | 2.33(1.98) | 1.17(1.35) | 1.81(2.16) | 0.88(1.04) |
| 5-10 | 3.00(2.14) | 2.64(1.97) | 2.28(2.08) | 1.02(1.34) | 1.84(2.07) | 1.12(1.08) |
| 10-18 | 3.64(1.67) | 2.77(2.00) | 2.47(1.89) | 1.36(1.45) | 1.89(1.79) | 1.17(0.892) |
| 18> | 2.85(1.73) | 2.20(1.98) | 2.30(2.10) | 0.70(1.13) | 1.65(2.20) | 0.93(0.904) |

**Boldness** statistically significant differences, DLQI Dermatology Life Quality Index Questionnaire, Sup Insurance Supplementary Insurance, SD Standard deviation, Other treatments refer to non-biologic therapies, including targeted systemic agents and topical treatments.
